# Evaluation of the External Soft Tissue Changes Following the Correction of Class II Skeletal Malocclusion in the Adolescence Period Using Removable and Fixed Functional Appliances: A Systematic Review and Meta-Analysis

**DOI:** 10.7759/cureus.57070

**Published:** 2024-03-27

**Authors:** Dima M Almrayati, Mohammad Y Hajeer, Alaa Oudah A Almusawi, Samer T Jaber, Mohammad O Baba, Mowaffak A Ajaj

**Affiliations:** 1 Department of Orthodontics, Damascus University, Damascus, SYR; 2 Department of Orthodontics, University of Al-Knooz, Basrah, IRQ; 3 Department of Orthodontics, Al-Wataniya Private University, Hama, SYR; 4 Department of Oral Medicine, University of Aleppo, Aleppo, SYR

**Keywords:** dynamax, twin-block appliance, skeletal class ii division 1 malocclusion, class ii division 1 malocclusion, functional treatment, esthetic line of ricketts, nasolabial angle, labiomental angle, labiomental fold, soft tissue changes

## Abstract

In this systematic review, we aimed to assess the current evidence regarding the effectiveness of functional treatment with both removable and fixed appliances to normalize the external soft tissue for skeletal class II adolescent individuals.

We performed a broad electronic search to retrieve relevant studies from nine databases to identify randomized controlled trials (RCTs) and controlled clinical trials (CCTs) that investigated soft tissue changes following functional treatment and evaluated the changes using 2D lateral cephalometric radiographs and 3D-optical surface laser scanning. A total of three RCTs and eight CCTs were included. Ages ranged from 11 to 16 years with the fixed functional appliances, and from eight to 12 years with the removable ones, including 689 skeletal class II patients. Version 2 of Cochran's risk-of-bias (RoB2), and the risk of bias in non-randomized studies of interventions (ROBIN-I) were used to assess the risk of bias for the included papers.

Of the 11 eligible studies, three studies were included in the meta-analysis to assess the upper and lower lip position in relation to the E-line (Ricketts's aesthetic line) in addition to the nasolabial angle. The meta-analysis showed that the upper lip retracted after functional treatment with Twin-block in relation to E-line (mean difference (MD) = -1.93; 95% CI: -2.37, -1.50; p < 0.00001; χ² = 5.43; p = 0.07; I^2^ = 63%), while the lower lip position did not change after functional treatment with Twin-block in relation to E-line (MD = 0.03; 95% CI: -0.56, 0.61; p = 0.92; χ² = 1.74; p = 0.42; I^2^ = 0%). The nasolabial angle increased after Twin-block treatment (MD = 5.75; 95% CI: 4.57, 6.93; p < 0.00001; χ² = 6.77; p = 0.03; I^2^ = 70%). The mentolabial angle and Z-angle also increased after functional therapy, where the facial convexity angle decreased, regardless of the functional devices used. On the other hand, using the 3D-optical surface laser scanning showed that the upper lip length and the commissural width did not change following therapy, but the lower lip increased in length, as well as the total face height. More high-quality RCTs are required to obtain accurate evidence in this field.

## Introduction and background

Skeletal class II is one of the most prevalent malocclusions orthodontists see in their practice. This type of malocclusion affects approximately 15% to 20% of the world's population [1]. According to Angle's classification, class II patients are frequently divided into division 1 and division 2 groups. Several causes can lead to the development of skeletal class II malocclusion, including genetic, environmental, and functional disorders. Among these factors, mandibular retrognathism is considered the most frequent diagnosis [2]. As a result, several treatment modalities are available for correcting class II deformities, including growth modification, camouflage treatment (with or without extraction), and surgical correction. Options for treatment vary with the type and severity of the malocclusion, the patient's age, and the pattern of facial development [3].

Growth modification is the best treatment modality for growing individuals [4]. It depends on putting the mandibular in a forward position. The muscles and soft tissues are stretched, and this force is generated and transmitted to the skeletal and dental structures, causing skeletal growth modification and tooth movement [5]. Functional orthopedic treatment can be accomplished using fixed or removable functional appliances [6].

The functional treatment results in a combination of dental and skeletal effects [7]. Skeletally, it causes an increase in the mandible length, a restriction on the maxilla, and growth in the condyle. Dentoalveolar alterations may account for as much, if not more, than the skeletal effects [8]. And it differs according to the appliance design [9]. With fixed appliances, the changes are mainly dentoalveolar compared with removable appliances [10]. The direct effects of hard tissue changes on soft tissues can be summarized by the advancement of the lower lip and the chin point and an improvement in facial profile [2]. However, other studies have found no change in all patients' profiles due to class II functional appliance treatment, and individual differences can be noted [10].

Many systematic reviews have found positive changes in the soft tissue drape following functional treatment. These include advancing the chin point, normalizing the lip relationship, and reducing facial convexity [11,12]. On the contrary, other systematic reviews have found no changes in the anteroposterior position of the lower lip position [13,14]. Moreover, the previous systematic reviews have been limited to evaluating a specific type of functional appliance design, whether removable or fixed, and there has been no systematic review encapsulating the two kinds of functional designs [10,15]. There is still a lack of evidence about external soft tissue changes following the functional treatment [9]. This systematic review summarises evidence from randomized controlled trials (RCTs) and controlled clinical trials (CCTs) on external soft tissue changes after correcting skeletal class II malocclusion with removable or fixed functional appliances during adolescence.

## Review

Materials and methods

Eligibility Criteria

The inclusion and exclusion criteria were used following the Population, Intervention, Comparison, Outcomes, and Study (PICOS) framework (See Appendices). Regarding participants, healthy growing patients of both genders with skeletal class II malocclusion and at the pubertal growth spurt for those treated with removable functional appliance and during or post-pubertal growth for those treated with fixed functional appliance regardless of a racial group were included. Concerning interventions, the functional orthopedic treatment with either removable or fixed functional appliances was included. Regarding the comparison groups, patients treated with a functional orthopedic appliance were different from those used in the experimental group or untreated patients. Regarding study designs, RCTs and CCTs were sought. The publications in all languages until December 2023 were accepted for inclusion. Finally, the outcome measures included variables evaluating external soft tissue changes assessed using lateral cephalometric radiographs or three-dimensional (3D) imaging methods. Case reports, or case series reports, retrospective studies, in-vitro studies, animal studies, editorial articles, personal opinions, studies that did not clearly describe the included sample, articles describing the therapeutic technique, and studies that assessed changes using solely electromyographic analyses were excluded.

Search Strategy

An electronic search was conducted using PubMed®, Medline® (Medical Literature Analysis and Retrieval System Online), Web of Science®, Scopus®, Embase® (Excerpta Medica Database), EBSCO (Elton B. Stephens Company), Google™ Scholar, the Cochrane Central Register of Controlled Trials (CENTRAL), and OpenGrey. To find papers published up until January 2023, in addition to the electronic search, the Angle Orthodontist, the American Journal of Orthodontics and Dentofacial Orthopedics, the European Journal of Orthodontics, the Journal of Orthodontics and Craniofacial Research, and the Journal of Orthodontics were manually searched. ClinicalTrials and the World Health Organisation International Clinical Trials Registry Platform Search Portal (ICTRP) were also used for all clinical studies that were completed or were in process or have not yet been published. More details about the search strategy used in databases and journals are described in Table 1.

**Table 1:** Electronic search strategy used in the current systematic review

Database	Search strategy
CENTRAL (The Cochrane Library)	#1 "class II malocclusion " OR " skeletal class II" OR "distal occlusion" OR "mandibular retrognathia" OR " mandibular retrognathism" #2 "growth modification" OR "functional treatment" OR "functional orthopedic" OR "jaw relationship correction" OR " mandibular advancement" OR "mandibular enlargement" OR " mandibular protrusion" OR "maxillary restriction" OR "mandibular protrusion appliance " OR "removable functional appliance" OR" fixed functional appliance" OR " Activator" OR "Frankle regulator OR "Bionater" OR "Twin block" OR "Herbst" OR "modified Herbst" OR "Hotz" OR "trainers " OR " Double plates" OR "Dynamax" OR "Miniblock" #3 "Soft tissue " OR "Soft-Tissue " OR" lip profile " OR " Facial profile" OR " Ricketts line" OR " E-line" OR " Merrifield’s line" OR" Holdaway’s line " OR " Steiner’s line" OR "facial convexity" OR "nasolabial angle OR "mentolabial angle" OR "H angle" OR "Z angle" OR "chain position" OR "upper lip position" OR "lower lip position" #4 #1 AND #2 AND #3
Embase	#1 "class II malocclusion " OR " skeletal class II " OR "distal occlusion" OR "mandibular retrognathia" OR " mandibular retrognathism" #2 "growth modification" OR "functional treatment" OR "functional orthopedic" OR "jaw relationship correction" OR " mandibular advancement" OR "mandibular enlargement" OR " mandibular protrusion" OR "maxillary restriction" OR "mandibular protrusion appliance " OR "removable functional appliance" OR" fixed functional appliance" OR " Activator" OR "Frankle regulator OR "Bionater" OR "Twin block" OR "Herbst" OR "modified Herbst" OR "Hotz" OR "trainers " OR "Double plates" OR "Dynamax" OR "Miniblock" #3 "soft tissue " OR "soft-tissue " OR" lip profile " OR " Facial profile "OR " Ricketts line" OR "E-line" OR " Merrifield’s line" OR" Holdaway’s line " OR " Steiner’s line" OR "facial convexity" OR "nasolabial angle " OR "mentolabial angle " OR "H angle" OR "Z angle" OR "chain position" OR "upper lip position" OR "lower lip position" #4 #1 AND #2 AND #3
PubMed	#1 "class II malocclusion " OR " skeletal class II" OR "distal occlusion" OR "mandibular retrognathia" OR " mandibular retrognathism" OR #2 "2 "growth modification" OR "functional treatment" OR "functional orthopedic" OR "jaw relationship correction" OR " mandibular advancement" OR "mandibular enlargement" OR " mandibular protrusion" OR "maxillary restriction" OR "mandibular protrusion appliance " OR "removable functional appliance" OR" fixed functional appliance" OR " Activator" OR "Frankle regulator OR "Bionater" OR "Twin block" OR "Herbst" OR "modified Herbst" OR "Hotz" OR "trainers " OR "Double plates" OR "Dynamax" OR "Miniblock" #3 "soft tissue " OR "soft-tissue " OR" lip profile " OR " Facial profile "OR " Ricketts line" OR "E-line" OR " Merrifield’s line" OR" Holdaway’s line " OR " Steiner’s line" OR "facial convexity" OR "nasolabial angle " OR "mentolabial angle " OR "H angle" OR "Z angle" OR "chain position" OR "upper lip position" OR "lower lip position" #4 #1 AND #2 AND #3
Scopus	#1TITLE-ABS-KEY ("class II malocclusion " OR " skeletal class II" OR "distal occlusion" OR "mandibular retrognathia" OR " mandibular retrognathism" OR ) #2TITLE-ABS-KEY ("growth modification" OR "functional treatment" OR "functional orthopedic" OR "jaw relationship correction" OR " mandibular advancement" OR "mandibular enlargement" OR " mandibular protrusion" OR "maxillary restriction" OR "mandibular protrusion appliance " OR "removable functional appliance" OR" fixed functional appliance" OR " Activator" OR "Frankle regulator OR "Bionater" OR "Twin block" OR "Herbst" OR "modified Herbst" OR "Hotz" OR "trainers " OR "Double plates" OR "Dynamax" OR "Miniblock ") #3TITLE-ABS-KEY ("soft tissue " OR "soft-tissue " OR" lip profile " OR " Facial profile "OR " Ricketts line" OR "E-line" OR " Merrifield’s line" OR" Holdaway’s line line " OR " Steiner’s line" OR "facial convexity" OR "nasolabial angle " OR "mentolabial angle " OR "H angle" OR "Z angle" OR "chain position" OR "upper lip position" OR "lower lip position" #5 #1 AND #2 AND #3
Web of Science	#1 TS= ("class II malocclusion " OR " skeletal class II" OR "distal occlusion" OR "mandibular retrognathia" OR " mandibular retrognathism" OR ) #2 TS= ( "growth modification" OR "functional treatment" OR "functional orthopedic" OR "jaw relationship correction" OR " mandibular advancement" OR "mandibular enlargement" OR " mandibular protrusion" OR "maxillary restriction" OR "mandibular protrusion appliance " OR "removable functional appliance" OR" fixed functional appliance" OR " Activator" OR "Frankle regulator OR "Bionater" OR "Twin block" OR "Herbst" OR "modified Herbst" OR "Hotz" OR "trainers " OR "Double plates" OR "Dynamax" OR "Miniblock(" #3 TS= ("soft tissue " OR "soft-tissue " OR" lip profile " OR " Facial profile“ OR " Ricketts line" OR "E-line" OR " Merrifield’s line" OR" Holdaway’s line " OR " Steiner’s line" OR "facial convexity" OR "nasolabial angle " OR "mentolabial angle" OR "H angle" OR "Z angle" OR "chain position" OR "upper lip position" OR "lower lip position(" #5 #1 AND #2 AND #3
Google Scholar	#1")class II malocclusion " OR " skeletal class II" OR "distal occlusion" OR "mandibular retrognathia" OR " mandibular retrognathism" OR ) AND ) "growth modification" OR "functional treatment" OR "functional orthopedic" OR" jaw relationship correction" OR " mandibular advancement" OR "mandibular enlargement" OR " mandibular protrusion" OR "maxillary restriction" OR "mandibular protrusion appliance "OR "removable functional appliance" OR" fixed functional appliance" OR " Activator" OR "Frankle regulator OR "Bionater" OR "Twin block" OR "Herbst" OR "modified Herbst" OR "Hotz" OR "trainers " OR "Double plates" OR "Dynamax" OR "Miniblock ") AND ")soft tissue " OR "soft-tissue " OR" lip profile " OR " Facial profile "OR " Ricketts line" OR "E-line" OR" Merrifield’s line" OR" Holdaway’s line " OR " Steiner’s line" OR "facial convexity" OR "nasolabial angle " OR "mentolabial angle " OR "H angle" OR "Z angle" OR "chain position" OR "upper lip position" OR "lower lip position" )
OpenGrey	#1 functional treatment or mandibular advancement AND soft tissue change OR lip profile change
World Health Organization (WHO) International Clinical Trials Registry Platform (ICTRP)	(Functional orthodontic treatment or functional orthopedic treatment or mandibular advancement or functional appliance or mandibular protrusion appliance) AND (soft tissue changes OR facial change)
ClinicalTrials.gov	(Functional orthodontic treatment or functional orthopedic treatment or mandibular advancement or functional appliance or mandibular protrusion appliance) AND (soft tissue changes OR facial change)

Study Selection

Two team members (DMM, MYH) independently assessed the studies and looked into their eligibility to be included in this systematic review. When the dispute occurred, a third author (AOAA) helped in resolving it.

In the beginning, only titles and abstracts were evaluated for each study, and when there was a possibility of inclusion, the full text was reviewed, in addition to studies whose title and summary were insufficient to make a decision. The information was also extracted by the same two researchers and the third reviewer, who was consulted in case of a dispute between the researchers. The data summary tables included the following items: general information (the name of authors, the year of publication, and study setting); methods (study design and treatment comparison); participants (sample size, age, and gender); intervention (the type of construction bite was taken); orthodontic aspects (malocclusion characteristics and period of active treatment), and outcomes.

Assessment of the Risk of Bias in the Included Studies and the Quality of Evidence

Cochrane's Risk of Bias tool 2.0 (ROB-2) tool for randomized trials was used by two reviewers (DMM, MYH) to assess the quality of the chosen articles [16]. The following domains of bias for randomized trials were rated as low, high, or some concern: bias arising from the randomization process, bias due to deviations from intended interventions (effect of assignment to intervention; effect of adhering to intervention), bias due to missing outcome data, bias in outcome measurement, and bias in the selection of the reported result. In the case of a disagreement, a third reviewer (AOAA) was consulted. The same reviewers used the Risk of Bias in Non-randomized Studies - of Interventions (ROBINS-I) tool for non-randomized studies [17]. The quality of evidence was assessed using the Grading of Recommendations, Assessment, Development, and Evaluation (GRADE) approach [18].

Results

Study Selection and Inclusion in the Review

**Figure 1:**
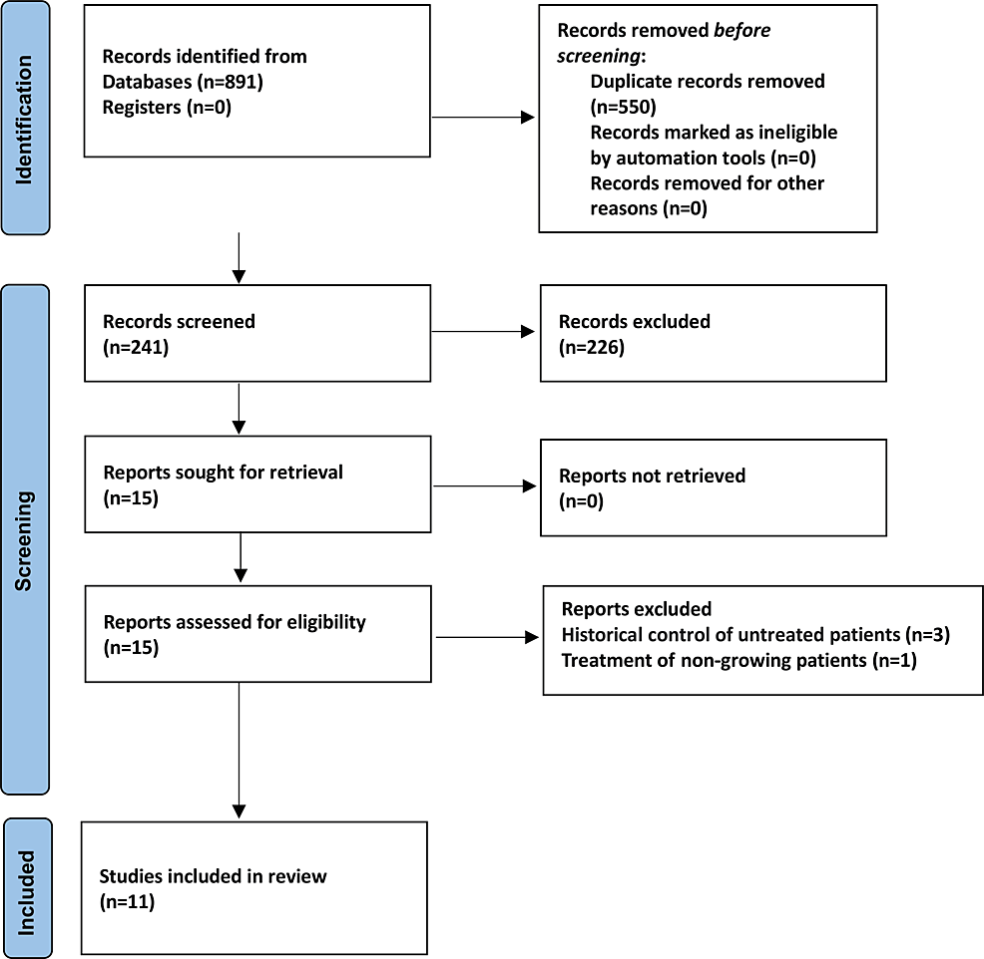
Preferred Reporting Items for Systematic Reviews and Meta-Analyses (PRISMA) flow diagram of the studies' identification, screening, and inclusion into this review

Figure 1 shows the flow chart of the studies chosen to be included in this study. Eight hundred ninety-one studies were identified from the electronic databases, and duplicate studies were eliminated. Two hundred forty-one studies' titles and summaries were reviewed; the text of 15 potentially relevant papers was revised. Four studies did not meet the inclusion criteria for this review, and the reasons for rejection are given in Table 2. Finally, this systematic review included 11 studies and three in the quantitative synthesis (i.e., the meta-analysis).

**Table 2:** Excluded studies and the reasons for exclusion

Authors, year	Study	Reason for exclusion
Franchi et al., 2011	Effectiveness of comprehensive fixed appliance treatment used with the Forsus Fatigue Resistant Device (FRD) in Class II patients	The experimental group was compared with a historical control group of untreated patients.
Oztoprak et al., 2012	A cephalometric comparative study of class II correction with Sabbagh Universal Spring (SUS) and Forsus FRD appliances	The treatment was provided to non-growing patients with a force fatigue-resistant appliance with a cervical skeletal maturity of CMVI5-CMVI6.
Landázuri et al., 2013	Changes in facial profile in the mixed dentition, from natural growth and induced by Balters' bionator appliance	The control group was selected from the documentation files of the Burlington Growth Centre.
Hourfar et al., 2018	Soft tissue profile changes after Functional Mandibular Advancer or Herbst appliance treatment in class II patient	The experimental groups were compared with a historical control group of untreated patients.

Characteristics of the Included Studies

Three studies were RCTs, and eight were CCTs, with 689 patients of both genders. The ages of the included patients ranged from 11 to 16 years for the fixed functional appliances and from eight to 12 for the removable ones. Two studies did not give any information about sex distribution, while the other studies included both genders. Of the 11 studies, seven evaluated removable appliances, two evaluated fixed ones, and the other two compared removable appliances against fixed appliances. The Twin-block appliance was evaluated in four trials in which the comparison was made against the Dynamax appliance in two studies [19,20] and with the mini-block appliance (a modified appliance of Twin-block, which was incrementally advanced employing maxillary incisor torquing springs) in one study [21].

On the other hand, the pure effects of the Twin-block appliance were quantified against an untreated control group in three studies [22-24]. The Frankel appliance was evaluated in the study of Stamenković et al. [25], who compared these appliances with the Activator and the Hotz appliances. One study evaluated the Activator and the Functional Trainer by Idris et al. [26]. Otherwise, two studies evaluated the fixed functional appliances. One compared two fixed functional appliances: force fatigue-resistance device (FRD) and AdvanSync [27]. The other paper compared Herbst with changes produced by normal growth [28].

On the other hand, only two studies combined the fixed and removable appliances, Herbst and activator, which were compared with normal growth [29]. The construction bite was taken in a single step by advancing the mandible in all studies except for two, where the mandibular advancement was performed in two steps [22,23]. All studies used cephalometric radiographs to evaluate post-treatment soft tissue changes, and only three of these studies included additional 3D appraisal using laser scanning [19-21].

Regarding the linear measurements, the positions of the upper and lower lips were evaluated in seven studies [22-26,28,29], other than the chin position, which was included in only five studies [21-23,28,29]. The lengths of upper and lower lips were measured in three studies [19-21], while the total anterior face height, the lower anterior face height, and commissural width were only included in the three studies that used laser scanning [19-21]. For angular measurements, facial convexity was studied in three trials [22,26,27], the nasolabial angle was assessed in four studies [22-24,27], and the mentolabial angle was examined in four studies [22,24,26]. The Z angle was evaluated in two studies [23,24]. The characteristics of the 11 included trials are illustrated in Table 3.

**Table 3:** Characteristics of the included studies RCT: randomized clinical trial; CCT: controlled clinical trials; EXP: experimental group; UL: upper lip; LL: lower lip; FR I: Frankel regulator type I; HBT: Herbst; H: Hotz appliance; BB: Belters' Bionator type I; FMA: functional mandibular advancement, AC: activator appliance; T4K: myofunctional trainer system; B: bionator; TB: twin-block appliance; MB: mini block, FRD: Forsus fatigue-resistant device, M: male; F: female; H angle: the angle formed between soft tissue nasion, soft tissue pogonion, and labrale superius; Z angle: the angle formed between Frankfort plane and Ricketts line; T angle: the angle formed between the mouth tangent (the line passing through soft tissue pogonion and the subnasal point) and the vertical plane through the subnasal point.

Study/setting	Methods		Participants	Type of malocclusion	Interventions		Outcomes
	Study design	Treatment comparison	Patients n (M/F); age (years)		The type of construction bite was taken	Active treatment period "meantime within T1-T0"	
Sharma and Lee, 2005, United Kingdom (UK) [21]	CCT	TB vs. MB	Patients 70 (35/35); age 10-14	Skeletal class II relationship caused by mandibular retrognathia and 7 mm minimum overjet	NI	9 months (SD 1 month) the relapse phase was 3 months with no retainer	Gonial width UL length, position LL length, position total anterior face height, Commissural width tragus to ST pogonion facial convexity, Chin position lower facial height
Quintão et al., 2006, Brazil [23]	CCT	TB vs. control untreated group	Patients 38 (24/14); mean age 9.7	Skeletal class II relationship (ANB > 4 degrees) caused by mandibular retrognathia	TB: Stepwise mandibular advancement	12 months (SD 1 month)	Nasolabial angle, chin position, UL position, LL position, Z angle
Lee et al., 2007, UK [19]	CCT	TB vs. Dynamax	Patients 62 (36/26); mean age M: 11-14, F: 10-13	Skeletal class II relationship caused by mandibular retrognathia and 7 mm minimum overjet	TB: Single-step mandibular advancement	The active treatment was 9 months and the relapse phase was 3 after the appliance was removed and no retainer was placed	UL length, position LL length, position total face height lower face height, Commissural width tragus to ST pogonion
Varlık et al., 2008, Turkey [24]	CCT	TB vs. AC vs. control untreated group	Patients 75 (38 /37); TB: 25, AC: 25; control: 25; age: 11.9±0.16	Skeletal class II pattern (ANB > 4) caused by mandibular retrognathia	Single-step mandibular advancement	AC: 9 months TB: 8 months	Z angle, nasolabial angle, mentolabial angle, UL position, LL position
Baysal and Uysal, 2013, Turkey [22]	CCT	TB vs. HBT vs. control untreated group	Patients 60 (30/30); TB: 20, HBT: 20; control: 20; age: 12-13 ± 1 year	Skeletal class II pattern (ANB > 4) caused by mandibular retrognathia	HBT: Single-step mandibular advancement; TB: Stepwise mandibular advancement	HBT: 15.81 TB: 16.20 control:15.58	Facial convexity, H angle, nasolabial angle, mentolabial angle, UL position length, LL position length, chin position
Lee et al., 2014, UK [20]	CCT	TB vs. Dynamax	Patients 150; age M: 11-4, F: 10-13	Skeletal class II relationship caused by mandibular retrognathia and 7 mm minimum overjet	TB: Single-step mandibular advancement	15 months the relapse phase was 6 months with no retainer	UL length, position LL length, position total anterior face height lower facial height, Commissural width tragus to ST pogonion, tragus to sulcus inferius
Bilgiç et al., 2015, Turkey [29]	RCT	FRD vs. AC vs. control non-treated group	Patients 60 (34/26); FRD: 20, AC: 20; control 20; age: 11-14 years	Skeletal class II pattern (ANB > 4) caused by mandibular retrognathia	AC: Single-step mandibular advancement	5.6 ± 1.8 months	UL position, LL position, chin position
Stamenković et al., 2015, Serbia [25]	CCT	FR I vs. BB vs. H	Patients 60 (28/32); FR: 20, BB: 20 H: 20; mean age 9 years and 9 months	Skeletal class II (ANB angle > 4), caused by mandibular retrognathia	NI	18-24 months	T angle, H angle, LL position, UL position
Idris et al., 2019, Syria [26]	RCT	Activator vs. Trainer	Patients 54 (28/26); AC: 28, T4K: 26; age: 8-12 years	Skeletal Class II relationship (ANB > 4 degrees) caused by mandibular retrognathia	AC: Single-step mandibular advancement	12 months	facial convexity, UL position, LL position, nasolabial angle, mentolabial angle
Martina et al., 2020, Italy [28]	CCT	HBT VS. control non-treated group	Patients 44 (22/22); exp: 22, control: 22; age 11.6 ± 1.3 years	Skeletal class II (ANB angle > 4), caused by mandibular retrognathia		12 months	Nasal prominence position, UL position, LL position, chin position
Hemanth et al., 2023, India [27]	RCT	FRD vs. AdvanSync	Patients 16; age 11-16 years	Skeletal class II with mandibular retrognathia, overjet of at least 5 mm		7-8 months	Facial convexity, nasolabial angle, mentolabial sulcus

Risk of Bias of the Included Studies

**Figure 2:**
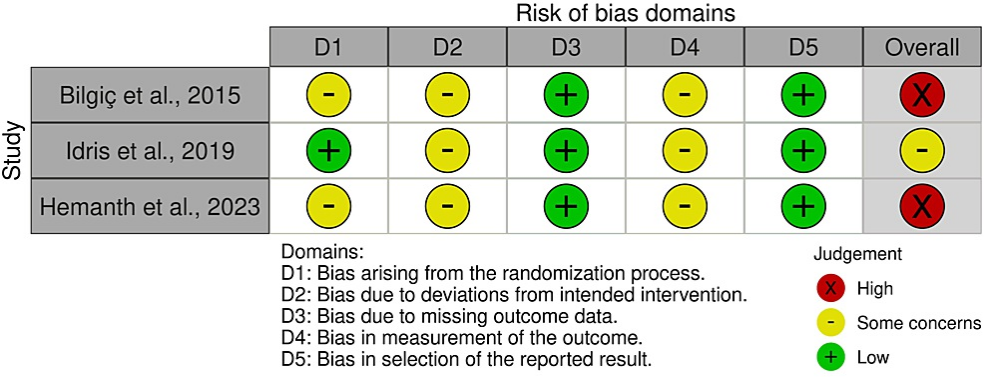
Risk of bias summary of the three randomized controlled trials (RCTs) Bilgic et al., 2015, [29]; Idris et al., 2019, [26]; Hemanth et al., 2023, [27]

**Figure 3:**
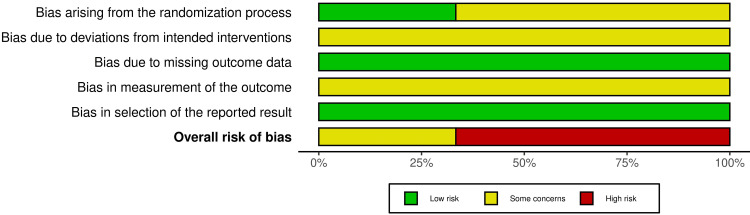
The overall risk of bias score for each field (or domain) for the included three randomized controlled trials (RCTs)

Figure 2 shows the risk of bias for the three included RCTs, while Figure 3 illustrates the overall risk of bias for every domain. See Appendices for more details related to the evaluation and supporting reasons. Two studies were assessed as "high risk of bias" [27,29], while the third RCT was at "some concerns" [26]. See Appendices for further information on the assessment and supporting reasons.

**Figure 4:**
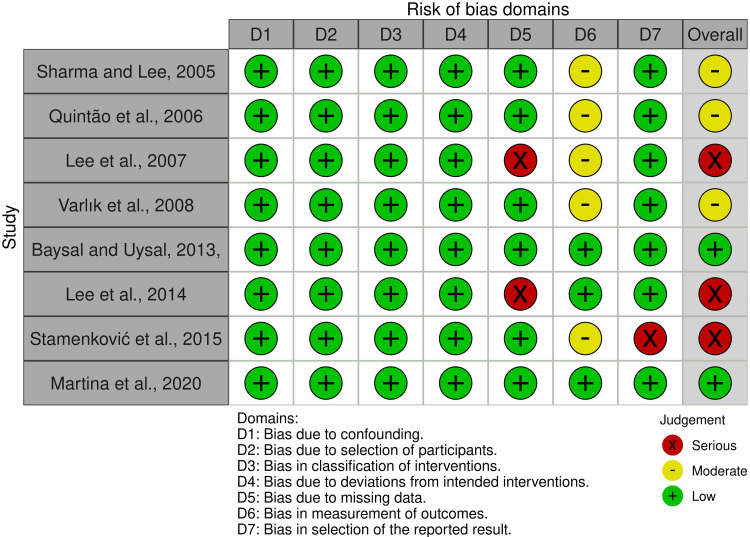
Risk of bias summary of the included controlled clinical trials (CCTs) Sharma and Lee, 2005, [21]; Quintao et al., 2006, [23]; Lee et al., 2007, [19]; Varlik et al., 2008, [24]; Baysal and Uysal, 2013, [22]; Lee et al., 2014, [20]; Stamenkovic et al., 2015, [25]; Martina et al., 2020, [28]

**Figure 5:**
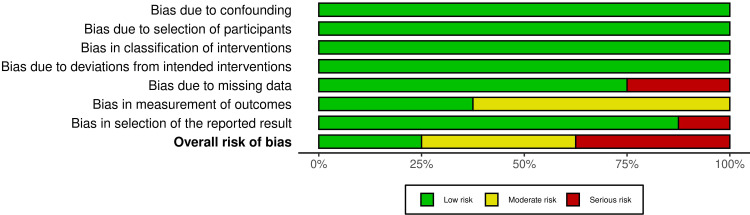
The overall risk of bias score for each field of the included controlled clinical trials (CCTs)

On the other hand, Figure 4 presents the risk of bias for the eight CCTs, and Figure 5 shows the overall risk for each field. Two CCTs were assessed as "low risk of bias" [22,28], three studies were at "moderate risk of bias" [21,23,24], and the other three CCTs were at "high risk of bias" [19,20,25]. See Appendices for further information on the assessment and supporting reasons.

Effects of Interventions

First: Positional changes of the lips and chin. These changes were only assessed on 2D lateral cephalometric radiographs.

**Figure 6:**
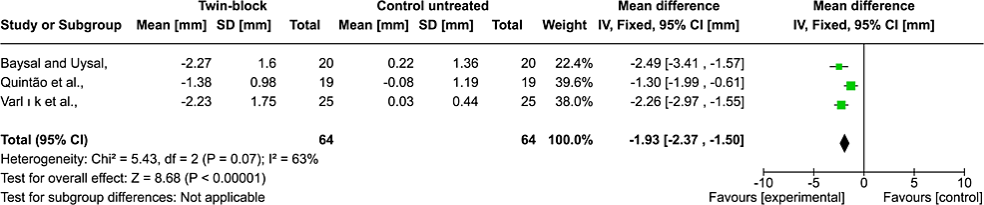
Forest plot of the comparison between the Twin-block and the untreated control groups regarding the upper lip position to E-line Baysal and Uysal, 2013, [22]; Quintao et al., 2006, [23]; Varlik et al., 2008, [24]

Upper lip position: Eight studies evaluated the position of the upper lip following functional treatment. Baysal and Uysal, Quintão et al., and Varlık et al. evaluated the upper lip position regarding the Ricketts esthetic line (E-line) after Twin-block treatment [22-24]. They found that the upper lip moved backward after treatment, and the pooled estimate showed that there was a statistically significant difference between the experimental groups and the untreated control group, and the heterogeneity between those three studies was high (MD = -1.93; 95% CI: -2.37, -1.50; p < 0.00001; χ² = 5.43; p = 0.07; I^2^ = 63%, Figure 6). In the Bilgiç et al. study, although the upper lip recession in the force FRD group was greater than in the activator one, the difference between the two groups was not statistically significant (x̅ = -1.58 ± 4.23, x̅ = - 0.34 ± 7.54, respectively [29]. Similarly, Idris et al. found that there was no statistically significant difference between groups (activator, trainer) regarding the upper lip recession (p = 0.585) [26]. Stamenković et al. found that the most prominent change in the upper lip position was associated with the use of the Frankel appliance, followed by Hotz, then by Bionator (-0.65 mm, -0.35 mm, and 0.15, respectively), but the significance of the differences was not reported [25]. On the contrary, Martina et al. evaluated changes following the use of the Herbst appliance compared to an untreated control group [28]. They reported that the upper lip protruded in both groups with no statistically significant difference between them (x̅ = 2.1 ± 2.1, x̅ = 1.8 ± 3.6, p =0.724, respectively). According to the GRADE guidelines, the evidence strength regarding the upper lip position to the E-line was very low. The overall quality of evidence for these outcomes, according to GRADE, is illustrated in Table 4.

**Table 4:** Summary of the findings according to the GRADE guidelines for the included trials High quality: Further research is very unlikely to change our confidence in the estimate of effect. Moderate quality: Further research is likely to have an important impact on our confidence in the estimate of effect and may change the estimate. Low quality: Further research is very likely to have an important impact on our confidence in the estimate of effect and is likely to change the estimate. Very low quality: We are very uncertain about the estimate. CI: confidence interval; MD: mean difference; CCT: controlled clinical trial; GRADE: Grading of Recommendations, Assessment, Development, and Evaluation; E-line: esthetic line, an imaginary line drawn from the nose tip to the chin a - decline one level for risk of bias; b - decline one level for risk of bias; c - decline one level for risk of bias and one level for imprecision

Quality assessment criteria							Summary of findings				Comments
Variable	No. of studies	Risk of bias	inconsistency	Indirectness	Imprecision	Other considerations	No. of patients	Effects		Certainty	
								Absolute (95% CI)	Relative (95% CI)		
Upper lip position to E-line	3 CCTs	Serious	Not serious	Not serious	Not serious	None	173	-	Mean -1.53 mm, CI 95%, -2.02, - 1.03	Very low ⊕⊖⊖⊖^a^	The difference between Twin-block and control untreated group was statistically significant.
Lower lip position to E-line	3 CCTs	Serious	Not serious	Not serious	Not serious	None	173	-	Mean 0.06 mm, CI 95%, -0.68, 0.79	Very low ⊕⊖⊖⊖^b^	The difference between Twin-block and control untreated group was not statistically significant.
Nasolabial angle change	3 CCTs	Serious	Not serious	Not serious	Serious	None	173	-	Mean 5.75°, CI 95%, 4.57, 6.93	Very low ⊕⊖⊖⊖^c^	The difference between Twin-block and control untreated group was statistically significant.

Upper lip length: Lee et al., in their two published trials that compared the Twin-block and the Dynamax appliances, found that the upper lip length did not change following the functional treatment, with no significant difference between the two groups (p = 0.684, p = 0.747, respectively) [19,20].

**Figure 7:**
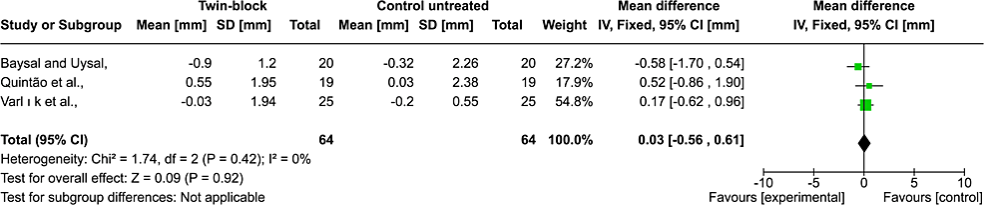
Forest plot of the comparison between the Twin-block and the untreated control groups regarding the lower lip position to E-line Baysal and Uysal, 2013, [22]; Quintao et al., 2006, [23]; Varlik et al., 2008, [24]

Lower lip position: The studies of Baysal and Uysal, Quintão et al., and Varlık et al. evaluated the lower lip position in relation to the E-line after Twin-block treatment. The pooled estimate showed that there was no statistically significant difference between the experimental group and untreated control group, and the heterogeneity between those three studies was low (MD = 0.03; 95% CI: -0.56, 0.61; p= 0.92; χ² = 1.74; p = 0.42; I^2^ = 0%, Figure 7) [22-24]. Similarly, there was no statistically significant difference between the two experimental groups in the study of Idris et al. (activator vs. trainer; p = 0.822), where the mean changes were x̅ = -0.05 ± 1.43, x̅ = 0.09 ± 1.52, respectively [26]. On the contrary, three studies showed statistically significant differences in the lower lip position among the studied groups. Martina et al. showed a greater increase in lower lip protrusion in the Herbst appliance group than in the untreated control one, with a clear significant difference between them (x̅ = 4.3 ± 3.1, x̅ = 2.1 ± 3.4, p = 0.030) [28]. Bilgiç et al. found in their study that the lower lip significantly protruded in both treatment groups (FRD and activator), and there was no statistically significant difference between groups (x̅ = 1.1 ± 3.68, x̅ = 1.91 ± 7.97, p = 0.786) [29]. Stamenković et al. discovered that treatment with Frankel and Bionator was associated with lower lip protrusions about 1 mm and 0.85 mm, respectively, while the Hotz appliance treatment retracted the lower lip by 0.20 mm [25]. 

Lower lip length: The first and second papers by Lee et al. reported that lower lip length increased after treatment with the Twin-block and the Dynamax appliances, and the largest increase was found in the Twin-block group (median change = 2.96, x̅ = 5.017 ± 5.093, respectively) compared with Dynamax group (median change = 0.98, x̅ = 2.917 ± 3.528, respectively). However, the first paper reported a statistically significant difference between groups, while the second paper reported no difference (p = 0.064, p = 0.001, respectively) [19,20].

Chin position: The results related to the change in the chin position were inconsistent among the retrieved studies. No significant change was seen between the untreated control and Twin-block groups (p = 0.605) regarding the chin position in the study of Quintão et al. [23]. Similarly, the study of Martina et al. also reported no statistically significant difference between the Herbst group and the untreated control one (p = 0.173) [28]. On the contrary, Baysal and Uysal reported a significant chin advancement in the Twin-block group, with a significant difference between the Twin-block group and the untreated control one (x̅ = 5.45 ± 3.8, x̅ = 1.95 ± 2.86, respectively) [22]. In addition, the study of Bilgiç et al. reported a significant difference between the FRD and the Activator group, where the most significant prominence was in the Activator group (x̅ = 2.13 ± 3.82, x̅ = 3.79 ± 7.22, p < 0.05, respectively) [29]. Shamra and Lee found that there was a statistically significant difference between the two experimental groups (Twin-block and Mini-block), and the greatest advancement was obtained with the Twin-block group (median change = 4, median change = 1.8, respectively, p = 0.004) [21].

Second: Change in certain angular measurements.This part covers four main variables: facial convexity, nasolabial, mentolabial, and Z angles.

Facial convexity change: Three studies included in this review evaluated the changes in facial convexity following functional therapy. Baysal and Uysal found a statistically significant difference between the Twin-block and the untreated control group. However, the Twin-block group had the largest increase in facial convexity angle, resulting in an improvement in the soft tissue profile (x̅ = 4.02 ± 2.46, x̅ = 0.12 ± 2.42, p < 0.0001, respectively) [22]. As well, Idris et al. showed in their results that the facial convexity angle increased in both groups (activator, trainer); the largest increase was found in the activator group compared with the trainer group (x̅= 2.61 ± 3.71, x̅ = 0.02 ± 2.51, p = 0.004, respectively) [26]. Hemanth et al. showed that there was a slight increase in facial convexity angle in both experimental groups (FRD, AdvanSync), with no statistically significant difference between the two groups (x̅ =1.316, x̅ =2.403, p = 0.30, respectively) [27].

**Figure 8:**
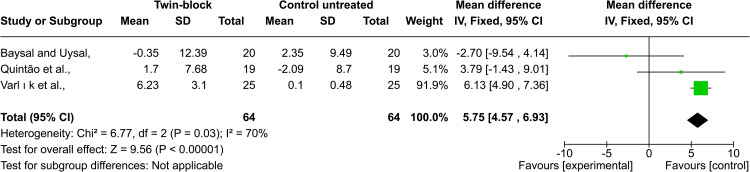
Forest plot of the comparison between the Twin-block and the untreated control groups regarding the nasolabial angle Baysal and Uysal, 2013, [22]; Quintao et al., 2006, [23]; Varlik et al., 2008, [24]

Nasolabial angle: Baysal and Uysal, Quintão et al., and Varlık et al. [22-24] evaluated the nasolabial angle change after Twin-block treatment. Varlık et al. reported that the nasolabial angle increased after treatment. In the other two studies by Baysal and Uysal, Quintão et al., the nasolabial angle did not significantly change after treatment. The pooled analysis showed that there was a statistically significant difference between the Twin-block group and the untreated control one, and the heterogeneity between those three studies was high (MD = 5.75; 95% CI: 4.57, 6.93; p < 0.00001; χ² = 6.77; p = 0.03; I^2^ = 70%, Figure 8) However, Hemanth et al., found in their study that there was no statistically significant difference between the two experimental groups (FRD, AdvanSync) (p = 0.12) [27]. According to the GRADE guidelines, the strength of evidence regarding nasolabial was very low. 

Mentolabial angle: Despite the different functional appliances studied in this review, the results of the three studies that examined mentolabial angle change after functional therapy showed an important increase in this angle. Varlık et al. reported a statistically significant increase in mentolabial angle in the Twin-block group compared to the untreated control group (x̅ = 16.35 ± 15.95, x̅ = 0.50 ± 1.45, p ≤ 0.001, respectively) [24]. Similarly, the study of Baysal and Uysal reported a statistically significant increase in Twin-block compared to the control untreated one (x̅ = 22.6 ± 13.27, x̅ = -10 ± 9, p < 0.001, respectively) [22]. Furthermore, Idris et al. found a significant increase in mentolabial angle in both groups: activator and trainer (x̅ = 11.96 ± 12.57, x̅ ®= 6.44 ± 14.77, respectively), and there was no statistically significant difference between the two groups [26].

Z-angle: Two studies in this review dealt with the changes in the Z-angle. The first one was done by Varlık et al., where they reported a statistically significant increase in the Twin-block group compared to the untreated control group (x̅ = 5.68 ± 6.50, x̅ = 0.28 ± 0.82, p ≤ 0.001, respectively) [24]. Furthermore, the study of Quintão et al. showed a statistically significant increase in the Twin-block group vs. the untreated control group ( x̅ = 2.79 ± 2.95, x̅ = -0.14 ± 3.86, p < 0.01) [23].

Third: Other changes assessed by 3D-optical surface laser scanning.

Commissural width: The commissural width change after treatment was assessed in three papers [19-21]. Shamra and Lee reported in their trial that the difference between the two experimental groups (Twin-block, Mini-block) was not statistically significant even at three months after the beginning of active treatment and after its nine-month ending [21]. The first paper by Lee et al., which compared Twin-block and Dynamax, reported no statistically significant difference between groups after the first six months of treatment (p = 0.311), whereas there was a difference between groups at the end of the active phase (nine months), and the largest increase was in the Twin-block group (median change = 2.88, median change= 0.75, p = 0.007, respectively) [19]. On the other hand, the second paper by Lee et al., which also compared Twin-block and Dynamax, reported no statistically significant difference between groups after the active treatment, which lasted 15 months (p = 0.305) [20].

Upper lip length: Three studies evaluated changes in upper lip length [19-21]. The study of Shamra and Lee reported no statistically significant difference between the Twin-block and Mini-block after three months of the treatment onset and the end of nine months of active treatment [21]. The first paper by Lee et al. showed a significant increase in upper lip length with the Twin-block appliance compared to the Dynamax appliance only at the end of nine months of treatment (median change = 1.45, median change = 0.05; p = 0.013, respectively) [19]. The second paper by Lee et al. found that there was no statistically significant difference between the Twin-block and Dynamax appliances following the completion of the 15-month treatment (p = 0.876) [20].

Lower lip length: Three studies reported no statistically significant change in the length of the lower lip during the treatment. Shamra and Lee found no statistically significant difference between groups after three months of onset and at the end of the nine-month active phase [21]. The results of the first paper by Lee et al. also showed no statistically significant difference even after six months and at the end of the active phase (p = 0.069, p =0.686, respectively) [19]. Similarly, the results of the second paper by Lee et al. also showed no statistically significant difference between groups after the active treatment (p = 0.519)[20].

Total facial high: The first paper by Lee et al. reported that there was a statistically significant increase in facial high in the Twin-block group compared to Dynamax after six months (median change = 2.31, median change = 0.4, p = 0.043, respectively) as well as at the end of active treatment (median change = 4.04, median change = 2.52, p = 0.026, respectively) [19]. Lee et al., in their second paper, showed that there was an increase in both groups (Twin-block, Dynamax), without a statistically significant difference between them (x̅ = 6.147 ± 4.395, x̅ = 6.094 ± 4.526, p = 0.9032, respectively) [20]. Shamra and Lee found no statistically significant difference between the Twin-block and the Mini-block throughout all active treatments [21].

Discussion

This systematic review seems to be the first to evaluate the evidence from RCTs and CCTs regarding the changes in facial soft tissues following treatment with fixed and removable functional appliances for adolescent patients with class II skeletal malocclusion. The evidence regarding the 2D and 3D changes reported in the retrieved studies was assessed.

First: Positional Changes of the Lips and Chin

Upper lip position: The meta-analysis revealed that using the twin block resulted in a posterior movement of the upper lip relative to the E-line (MD = -1.93) [22-24]. This change has been noted in other studies dealing with other types of functional devices. Bilgic et al. also found a comparable result after six months of treatment with FRD and activator. However, the recession with the FRD was greater and clinically more important (a mean of -1.58 mm and a mean of -0.34 mm, respectively) [29]. In addition, the study by Idris et al. reported that treatment with an activator and trainer caused a lower amount of upper lip retrusion (a mean of -0.60 mm, a mean of -0.30 mm, respectively), and it is considered clinically negligible [26]. This posterior movement of the upper lip can be attributed to adaptation to dentoalveolar and skeletal changes resulting from treatment. On the other hand, Martina et al. reported that the upper lip protruded after seven months of Herbst treatment (a mean of +2.1 mm), and they explained that these changes are most likely ascribed to the patient’s growth not caused by the appliance.

Lower lip position: The results of the meta-analysis showed no statistically or clinically significant change in the position of the lower lip after active treatment with Twin-block in relation to E-line (MD = +0.03) [22-24]. These results were close to those reported by other studies. Furthermore, Idris et al. reported that both the activator and the trainer do not change the position of the lower lip in relation to the E-line after active treatment [26]. However, the study by Martina et al. reported that the lower lip protruded after treatment with Herbst (a mean of +4.3 mm) [28]. As such, Bilgic et al. found the same change after treatment with an FRD and an activator (a mean of +1.1 mm and a mean of +1.91 mm, respectively) [29]. This dissimilarity is due to the difference in ways of evaluating. The studies that showed that the lower lip did not change were evaluated using the E-line. Ricketts's esthetic line shifted as the pronasale and soft tissue pogonion advanced following the treatment, so they reported that the lower lip did not change in relation to the shifting Ricketts line. The evaluation was done in relation to a fixed reference line in the study of Bilgic et al. and Martina et al., which indicated that the lower lip moved significantly in the anterior direction.

Chin position: According to the findings of studies that examined the amount of chin protrusion after functional treatment, there was a significant anterior movement after treatment with Twin-blocks (a mean of +4 mm) and activators (a mean of +3.7 mm), and the lower advancement was with Mini-blocks (a mean of +1.8 mm). On the other hand, the advancement with FRDs was less (a mean of +2.13 mm), but with the Herbst appliance, that advancement was clinically evident (a mean of +4.4 mm) [21-23,28,29]. In general, the chin forward movement can be explained by the increased length of the mandibular body resulting from functional treatment. As it's known, the effect of fixed functional appliances is mainly dentoalveolar, with a little skeletal change. The significant variation in previous studies prevented a quantitative synthesis of the results.

Second: Angular Measurements

Facial convexity: According to three studies in this hub [22,26,27], attractiveness changes in the facial soft tissue profile are reported in all three studies. The profile convexity decreased in the treated samples, introducing a straighter profile. The greatest increase was accompanied after treatment with Twin-blocks (a mean change of 4°), while treatment with activators caused a slight increase (a mean change of 2.6°), and trainers had the least increase (a mean change of 0.02°). On the other hand, the fixed devices FRD and AdvanSync introduced a slight improvement (a mean change of 1.3° and 2.4°, respectively). This change is mostly due to the increased mandibular length due to the functional treatment and pogonion advancement, other than the slight inhibitions on the maxilla. The significant variation in previous studies prevented a quantitative synthesis of the results.

Mentolabial angle: The three studies included in this review showed the mentolabial angle increased significantly after functional treatment. Twin-blocks resulted in a larger increase, according to the studies of Varlık et al., Baysal, and Uysal (a mean change of 16.3 and 22.6, respectively) [22,24]. While treatment with trainers resulted in a less significant increase (a mean change of 6.4°) [26]. This increase is primarily due to the effect of functional treatment upon reduction of overjet, which prevented the lower lip from being distorted and curled. An additional factor that may play a role is maintaining the lip seal while wearing the appliance, which increases lip strain and changes the tonicity and posture of the perioral muscles. 

Z-angle: According to the two studies in this domain, the Z-angle increased due to functional treatment with Twin-blocks [23,24]. This was primarily due to the pogonion's forward movement and a slight recession of the upper lip.

Nasolabial angle: According to the meta-analysis, the nasolabial angle increased due to functional treatment with Twin-blocks, and this change was due to the nasal base and upper lip retrusion (MD = 5.75°) [22-24]. However, Hemanth et al. found that this angle did not change after functional treatment with FRD and AdvanSync, and this can be explained by the fact that the fixed functional appliances caused changes, which are mainly dentoalveolar, and their effect on soft tissue is limited [27]. 

Third: Other Changes Assessed by 3D-Optical Surface Laser Scanning

Total facial height: There is agreement among the studies that facial height increased after Twin-block and Dynamax treatments, and Mini-block had a minimal amount of facial height increase due to its design [19-21]. This increase can be explained by the changes in vertical dimension caused by lower jaw advancement.

Lower lip length: According to studies included in this review, the lower lip length increased after Twin-block, Mini-block, and Dynamax treatment. This is primarily due to the absence of lip distortion following the labial competence normalizing and the reduction of overjet [19-21]. 

Upper lip length: All studies in this review agreed that the upper lip length did not change following treatment, whether using Twin-block, Mini-block, or Dynamax [19-21].

Commissural width: All studies in this review agreed that there was no important change in the commissural width following treatment, whether using Twin-block, Mini-block, or Dynamax [19-21].

Limitations of the current review

Only three RCTs and eight CCTs that met the eligibility criteria were identified and included in this systematic review (SR); three CCTs were at serious risk of bias, three CCTs were at moderate risk, and only two CCTS had a low risk of bias. On the other hand, two RCTs were considered at high risk of bias, while the third was determined to have some concern of bias. The strength of the evidence ranged from low to very low. As a result, high-quality RCTs are needed to accurately assess soft tissue changes following functional treatment with both removable and fixed appliances. The high heterogeneity, different types of functional appliances, and different evaluation methods prevented the inclusion of all the studies in the meta-analysis, and only the results of three studies were pooled.

## Conclusions

Despite the variability in reporting results, the included studies showed positive effects on facial soft tissues after treatment with removable and fixed functional appliances. The facial convexity and the mentolabial angle decreased, and the nasolabial angle increased, even with the variety of appliances used. Vertically, the facial height increased, as did the lower lip length. Regarding other parameters, variable results were found due to the variety of appliance designs and ways of evaluation. Since the available evidence ranges from low to very low, it seems necessary to conduct more RCTs to assess the changes resulting from functional treatment and find a more systematic and accurate protocol that evaluates the variables in relation to a fixed reference line that is unaffected by treatment or growth.

## Appendices

The Population, Intervention, Comparison, Outcomes, and Study (PICOS) framework exclusion and exclusion criteria are given in Table 5.

**Table 5:** The inclusion and exclusion criteria according to the PICOS framework PICOS: Population, Intervention, Comparison, Outcomes, and Study (PICOS)

Item	Inclusion criteria	Exclusion criteria
Participant characteristics	Healthy growing patients of both genders with skeletal class II malocclusion and at the pubertal growth spurt for those treated with removable functional appliance and during or post-pubertal growth for those treated with fixed functional appliance regardless of a racial group	Patients with craniofacial syndromes and/or cleft lip palate
		Patients with temporomandibular joint disorders/traumas/previous maxillofacial surgery
		Animal studies
Intervention	Functional orthopedic treatment with either removable or fixed functional appliance	Patients with Class II malocclusion treated with extractions, Class II elastics, orthognathic surgery, or previous orthodontic treatment
Comparison	Untreated patients with Class II malocclusion or functional orthopedic treatment with another appliance	The patient was treated with an extra-oral functional appliance
Outcome	external soft tissue changes assessed using lateral cephalogram radiograph and non-cephalometric assessment	
Study design	Randomized controlled clinical trials and controlled clinical trials	Unsupported opinion of expert
		Editor’s choices
		Replies to the author/editor
		Interviews
		Commentaries
		Books’/conferences’ abstracts
		Summaries
		Cross-sectional surveys
		Case series without a control
		Case reports
		Case-control observational studies
		Cohort studies
		Retrospective clinical trials
		Narrative reviews
		Systematic reviews
		Meta-analyses

The risk of bias assessment of the RCTs was performed using the RoB-2 tool as given in Table 6.

**Table 6:** Details of the risk of bias assessment of the randomized controlled trials using the RoB-2 tool RoB-2: Cochrane's Risk of Bias tool 2.0

Study	Bias arising from the randomization process	Bias due to deviations from intended interventions		Bias due to missing outcome data	Bias in the measurement of the outcome	Bias in the selection of the reported result	Overall bais
		Effect of assignment to intervention	Effect of adhering to intervention				
	D1	D2		D3	D4	D5	
Bilgiç et al., 2014 [29]	Some concerns: The method used for randomization was not reported. “The study group patients were randomly selected from patients who were admitted to our clinic.”	Some concerns: Blinding cannot be performed. There is no information on whether any deviations arose because of the trial context.	Some concerns: Blinding cannot be performed. There is no information on whether the important non-protocol interventions were balanced across intervention groups.	Low risk: No dropouts were reported.	Some concerns: No details of the blinding of outcome assessors were reported. The method of measuring the outcome was appropriate.	Low risk: No information about the registration protocol was mentioned. However, the reported outcomes in the result section seemed to correspond with the pre-defined outcomes aforesaid in the method section.	High risk
Idris et al., 2018 [26]	Low risk: “Randomization was performed by one of the academic staff at the Orthodontic Department who was not involved directly in this research project using a computer-generated random sequence of numbers.”	Some concerns: Blinding cannot be performed. There is no information on whether any deviations arose because of the trial context.	Some concerns: Blinding cannot be performed. There is no information on whether the important non-protocol interventions were balanced across intervention groups.	Low risk: Six patients (two patients in the activator group and four in the trainer group). “Six patients were lost to follow-up due to different reasons.” Nearly all the outcome data is available (90%).	Some concerns: “No blinding was applied in this trial.” The method of measuring the outcome was appropriate.	Low risk: The trial was not registered in any major database of clinical trials. However, the reported outcomes in the result section seemed to correspond with the pre-defined outcomes aforesaid in the method section.	Some concerns
Hemanth et al., 2023 [27]	Some concerns: The method used for randomization was not reported.	Some concerns: Blinding cannot be performed. There is no information on whether any deviations arose because of the trial context.	Some concerns: Blinding cannot be performed. And there is no information on whether the important non-protocol interventions were balanced across intervention groups.	Low risk: No dropouts were reported.	Some concerns: No details of blinding of outcome assessors were reported. The method of measuring the outcome was appropriate.	Low risk: No information about the registration protocol was mentioned. However, the reported outcomes in the result section seemed to correspond with the pre-defined outcomes aforesaid in the method section.	High risk

The ROBINS-I tool was used for the risk of bias assessment of non-RCTs as shown in Table 7.

**Table 7:** Details of the risk of bias assessment of the non-randomized clinical trials using the ROBINS-I tool. ROBINS-I: Risk of Bias in Non-randomized Studies - of Interventions

Study	Risk of bias due to confounding	Risk of bias in the selection of participants for the study	Risk of bias in the classification of interventions	Risk of bias due to deviations from intended interventions	Risk of bias due to missing data	Risk of bias in the measurement of outcomes	Risk of bias in the selection of the reported result	Over-all bias
Sharma and Lee, 2005 [21]	Low risk: No confounding expected	Low risk: All the participants at the start of follow-up and the start of intervention coincided	Low risk: Intervention was well-defined	Low risk: There were no deviations from the intended intervention beyond what would be expected in usual practice	Low risk: Data were available for all participants No dropouts were reported	Moderate risk: No information about outcome assessors blinding but the measurement repeated at another time	Low risk: The measurements and analysis of selected variables were reported in the study.	Moderate risk of bias
Quintão et al., 2006 [23]	Low risk: No confounding expected	Low risk: All the participants at the start of the follow-up and the start of the intervention coincided	Low risk: Intervention was well-defined	Low risk: There were no deviations from the intended intervention beyond what would be expected in usual practice	Low risk: Data were available for all participants	Moderate risk: No information about outcome assessors blinding	Low risk: The measurements and analysis of selected variables were reported in the study.	Moderate risk of bias
Lee et al., 2007 [19]	Low risk: No confounding expected	Low risk: All the participants at the start of the follow-up and the start of the intervention coincided	Low risk: Intervention was well-defined	Low risk: There were no deviations from the intended intervention beyond what would be expected in usual practice	Serious risk: Data were available for not all participants. “Six patients failed to complete the 12-month protocol”	Moderate risk: No information about outcome assessors blinding	Low risk: The measurements and analysis of selected variables were reported in the study.	Serious risk of bias
Varlık et al., 2008 [24]	Low risk: No confounding expected	Low risk: All the participants at the start of the follow-up and the start of the intervention coincided	Low risk: Intervention was well-defined	Low risk: The measurements and analysis of selected variables were reported in the study	Low risk: Data were available for all participants	Moderate risk: No information about outcome assessors blinding	Low risk: The measurements and analysis of selected variables were reported in the study.	Moderate risk of bias
Baysal and Uysal, 2013 [22]	Low risk: No confounding expected	Low risk: All the participants at the start of the follow-up and the start of the intervention coincided	Low risk: Intervention was well-defined	Low risk: There were no deviations from the intended intervention beyond what would be expected in usual practice	Low risk: Data were available for nearly all participants	Low risk: No information about outcome assessors blinding but the measurements reviewed twice by another investigator	Low risk: The measurements and analysis of selected variables were reported in the study	Low risk of bias
Lee et al., 2014 [20]	Low risk: No confounding expected	Low risk: All the participants at the start of the follow-up and the start of the intervention coincided	Low risk: Intervention was well-defined	Low risk: There were no deviations from the intended intervention beyond what would be expected in usual practice	Serious risk: Data were available for not all participants. “26 patients failed to wear appliances“	Low risk: The assessors were blinding	Low risk: The measurements and analysis of selected variables were reported in the study.	Serious risk of bias
Stamenković et al., 2015 [25]	Low risk: No confounding expected	Low risk: All the participants at the start of the follow-up and the start of the intervention coincided	Low risk: Intervention was well-defined	Low risk: There were no deviations from the intended intervention beyond what would be expected in usual practice	Low risk: Data were available for nearly all participants	Moderate risk: No information about outcome assessors blinding	Serious risk: There were no reports for all measurements and analysis of selected variables	Serious risk of bias
Martina et al., 2020 [28]	Low risk: No confounding expected	Low risk: All the participants at the start of the follow-up and the start of the intervention coincided	Low risk: Intervention was well-defined	Low risk: There were no deviations from the intended intervention beyond what would be expected in usual practice	Low risk: Data were available for nearly all participants	Low risk: The assessors were blinding	Low risk: The measurements and analysis of selected variables were reported in the study.	Low risk of bias
